# Use of compulsory community treatment in mental healthcare: An integrative review of stakeholders’ opinions

**DOI:** 10.3389/fpsyt.2022.1011961

**Published:** 2022-11-03

**Authors:** Dieuwertje Anna de Waardt, Anne Laura van Melle, Guy Antoine Marie Widdershoven, Wichor Matthijs Bramer, Franciscus Martinus Machiel Adrianus van der Heijden, Jorun Rugkåsa, Cornelis Lambert Mulder

**Affiliations:** ^1^Department of Psychiatry, ETZ Hospital (Elisabeth-TweeSteden Ziekenhuis), Tilburg, Netherlands; ^2^GGZ inGeest Mental Health Care, Amsterdam, Netherlands; ^3^Department of Ethics, Law, and Humanities, Amsterdam University Medical Centers (Location VUmc), Vrije Universiteit Amsterdam, Amsterdam, Netherlands; ^4^Medical Library, Erasmus MC, Erasmus University Medical Center, Rotterdam, Netherlands; ^5^Department Emergency Psychiatry, Vincent van Gogh for Psychiatry, Venray, Netherlands; ^6^Health Services Research Unit, Akershus University Hospital, Lørenskog, Norway; ^7^Centre for Care Research, University of South-Eastern Norway, Porsgrunn, Norway; ^8^Parnassia Psychiatric Institute, Rotterdam, Netherlands; ^9^Department of Psychiatry, Epidemiological and Social Psychiatric Research institute (ESPRi), Erasmus MC, Erasmus University Medical Center, Rotterdam, Netherlands

**Keywords:** involuntary treatment, attitude of health personnel, personal satisfaction, family, personal autonomy, outpatient compulsory treatment, supervised community treatment, community treatment order

## Abstract

**Background:**

Multiple studies have examined the effects of compulsory community treatment (CCT), amongst them there were three randomized controlled trials (RCT). Overall, they do not find that CCT affects clinical outcomes or reduces the number or duration of hospital admissions more than voluntary care does. Despite these negative findings, in many countries CCT is still used. One of the reasons may be that stakeholders favor a mental health system including CCT.

**Aim:**

This integrative review investigated the opinions of stakeholders (patients, significant others, mental health workers, and policy makers) about the use of CCT.

**Methods:**

We performed an integrative review; to include all qualitative and quantitative manuscripts on the views of patients, significant others, clinicians and policy makers regarding the use of CCT, we searched MEDLINE, EMBASE, PsycINFO, CINAHL, Web of Science Core Collection, Cochrane CENTRAL Register of Controlled Trials (via Wiley), and Google Scholar.

**Results:**

We found 142 studies investigating the opinion of stakeholders (patients, significant others, and mental health workers) of which 55 were included. Of these 55 studies, 29 included opinions of patients, 14 included significant others, and 31 included mental health care workers. We found no studies that included policy makers. The majority in two of the three stakeholder groups (relatives and mental health workers) seemed to support a system that used CCT. Patients were more hesitant, but they generally preferred CCT over admission. All stakeholder groups expressed ambivalence. Their opinions did not differ clearly between those who did and did not have experience with CCT. Advantages mentioned most regarded accessibility of care and a way to remain in contact with patients, especially during times of crisis or deterioration. The most mentioned disadvantage by all stakeholder groups was that CCT restricted autonomy and was coercive. Other disadvantages mentioned were that CCT was stigmatizing and that it focused too much on medication.

**Conclusion:**

Stakeholders had mixed opinions regarding CCT. While a majority seemed to support the use of CCT, they also had concerns, especially regarding the restrictions CCT imposed on patients’ freedom and autonomy, stigmatization, and the focus on medication.

## Introduction

Compulsory Community Treatment (CCT) is available as a coercive outpatient treatment option in many countries, including the USA, Canada, Australia, New Zealand, Asia, UK, and the Netherlands (1, 2). It is also known as Outpatient Compulsory Treatment or Supervised Community Treatment. The intention of this court-ordered treatment is to offer a less restrictive alternative to involuntary admission and to prevent relapses and the readmissions that can result from problems such as non-compliance with treatment. Although patients remain in the community, they have to comply with certain conditions such as taking medication or keeping appointments. The consequence of not complying with these conditions is usually readmission to a psychiatric hospital (3). In several countries, including United Kingdom, the court order is called a community treatment order (CTO).

There is an ongoing debate about the evidence on the effectiveness of CCT. Reviews of randomized controlled trials (RCTs) and pre-post studies on the effects of CCT did not demonstrate that CCT was more effective than voluntary outpatient care, either in reducing the number or duration of hospital admissions or in improving clinical outcomes (4, 5). The last Cochrane review in 2014 summarized the RCTs as follows: “CCT results in no significant difference in service use, social functioning or quality of life compared with standard voluntary care. […] However, [these] conclusions are based on three relatively small trials, with high or unclear risk of blinding bias, and low- to moderate-quality evidence” (5).

The most recent meta-analysis about the effects of CCT, states: “We found no consistent evidence that CCT reduces readmission or length of inpatient stay, although it might have some benefit in enforcing use of outpatient treatment or increasing service provision, or both” (4).

Kisely et al. performed a meta-analysis on outcomes of CCT in Australia and New Zealand. They did not find that CCT reduced the duration or number of admissions (6). Neither did the observational study of Weich et al. (7). There is some evidence suggesting that longer CTO’s are of greater benefit in improving outcome measures (6, 8). Other recent naturalistic studies did find that CCT increased treatment adherence, could increase the time people spent outside hospital, could decrease suicide risk and mortality and could decrease the duration of admission to hospital (8–11).

Despite discussions about its effectiveness, CCT is still used in many countries (4). This might be because in developing mental health laws, other views, factors, and experiences are taken into account. It may be that stakeholder groups (clinicians, patients, significant others, and policy makers) have positive views on the use CCT, despite a lack of scientific evidence of its effectiveness.

Corring et al. performed a constant comparative analysis of published qualitative research of three stakeholder groups (patients, relatives, and mental health workers) concerning CCT. They find that all three groups see benefits that outweigh the coercive nature of CCT, but also name limitations regarding the representativeness of people on the CTO group, which may bias the results (12).

With this integrative review we added to this knowledge by:

(1) Integrating both the qualitative as well as the quantitative results of studies on the views on CCT of these stakeholder groups, now also searching for the views of policy makers.

(2) Investigating whether their opinion was influenced by having experience with CCT.

## Methods

Integrative reviews – the method we chose to analyse the existing literature – were described by Whittemore and Knafl as “the broadest type of research review methods allowing for the simultaneous inclusion of experimental and non-experimental research in order to more fully understand a phenomenon of concern. [They] may also combine data from the theoretical as well as empirical literature” (13). By allowing for the inclusion of different methodologies (e.g., both quantitative and qualitative) to represent the current knowledge on a subject (13), integrative reviews are therefore very suited to analyse the wide-ranging literature on stakeholders’ views and experiences, as any restrictions on the inclusion of the manuscripts based on methodology would lead to the loss of valuable inputs.

Whittemore and Knafl describe five steps in performing an integrative review: (1) Problem identification, (2) Literature search, (3) Data evaluation, (4) Data analysis, and (5) Presentation.

These steps were followed in the execution of this integrative review.

### Problem identification

While there is no evidence from empirical studies (see “Introduction” section) that CCT is an effective way to reduce time spent in hospital, the number of admissions or to improve clinical outcomes, many countries still use this measure. Maybe this decision is based on opinions of stakeholders who have other arguments than scientific evidence to be in favor of a mental health system including CCT. Therefore, we would like to know: (1) the opinions of the various stakeholders (patients, significant others, mental health workers, and policy makers) on the use of CCT and whether their opinion was influenced by having experience with CCT; and (2) the advantages and disadvantages of CCT these stakeholders identified.

### Literature search

The following electronic bibliographic databases were searched two times, on 24 September 2019 and 27 August 2021 (date last searched) for manuscripts published in English: MEDLINE (via Ovid), EMBASE (via embase.com), PsycINFO (via Ovid), CINAHL (via EBSCOhost), Web of Science Core Collection, Cochrane Central Register of Controlled Trials (via Wiley) and Google Scholar. Although we used no filters for dates, populations and study designs, conference abstracts were removed from the search. Using the method described by Bramer et al. (14), the search was developed by an experienced information specialist (WMB) in close collaboration with the first author (DW). It consisted of four elements that are searched as controlled terms (MeSH or Emtree terms) and free text terms in title and/or abstract:

(1) Compulsory or involuntary, (2) outpatient or community, (3) mental health care or psychiatric diseases, and (4) experiences or opinion. We limited the results to articles published in the English language. Appendix 1 lists the search terms for all databases. References were imported in EndNote and deduplicated according to the method described by Bramer et al. (15).

### Data evaluation

AM and DW screened the title and, if the title indicated that the manuscript could be relevant, abstract of all the manuscripts in order to identify and include:

- All qualitative and quantitative studies on the views of patients, significant others (partner, family, and carers), clinicians and policy makers regarding the use of CCT.

In the selected manuscripts, we also checked all references for relevant studies. If there was no initial consensus on including the manuscript for full text reading, or if the title and abstract did not provide enough information to decide whether a manuscript should be included at this stage, the manuscript was selected for full-text reading.

DW and AM separately reviewed the manuscripts selected. Each manuscript was thoroughly read by both DW and LM separately to see if the authors described the opinion of the participants concerning whether or not they supported the use of CCT.

Table 1 describes the inclusion and exclusion criteria.

**TABLE 1:** Inclusion and exclusion criteria in the full text reading phase.

**Inclusion** - Quantitative and qualitative studies on the views of stakeholders regarding the use of CCT - Only manuscripts are included that express whether or not stakeholders support the use of CCT - Manuscripts published only in English in peer-reviewed journals until September 2021 **Exclusion** - Manuscripts that do not indicate whether stakeholders support or reject a system with CCT - Manuscripts that study the same population as another included manuscript (the manuscript that focused most on our question was chosen in these situation)

Then from the selected manuscripts the following data was extracted using a data extraction table:

- Which stakeholder groups.
- Whether the study used qualitative or quantitative methods.
- Country the study was performed in.
- In which way data was collected.
- Number of stakeholders.
- Whether or not participants had experience with CCT.
- For quantitative manuscripts: the percentages of stakeholders that were either for or against CCT.
- For qualitative manuscripts: terms in the studies that described the stakeholders’ majority view, such as “generally preferred…”, “supported”, “were opposed to”, “rejected” or “favored”. When possible, in the results of this review the literal phrases in the manuscripts are used to describe the results.

Discrepancies between DW and AM regarding the conclusion in qualitative manuscripts that the majority of the participants were in favor, were mixed or against the use of CCT, were discussed until consensus could be reached.

Quantitative results and qualitative results were summarized in a single table.

When the different stakeholder groups mentioned specific advantages or disadvantages of CCT, these were extracted and included in a separate table, being ranked from most to least mentioned by stakeholders in the different manuscripts.

No separate quality assessment of manuscripts was conducted. To ensure the quality of the manuscripts we only included manuscripts that had been published in journals with peer review.

## Results

The search in the different databases identified 5,300 manuscripts, from which 2,711 unique articles remained after deduplication. On the basis of their title and in some cases abstract, 2,569 of the identified manuscripts were excluded, as they did not meet our inclusion criteria.

**FIGURE 1:**
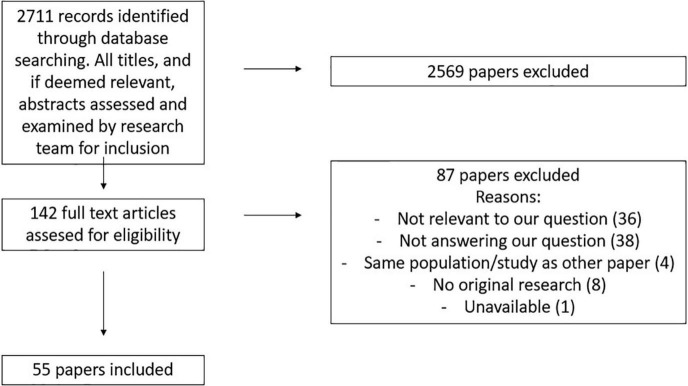
Literature selection process.

Finally, after full text screening, 55 manuscripts were included in the analysis (see Figure 1).

Table 2 lists the stakeholders’ opinions on the use of CCT. Quantitative outcomes are reported as percentages. The outcomes of qualitative studies are reported as they were reported in the manuscript. The number of participants named in the table for these quantitative studies, is, as far as it could be traced back, the number of participants answering the question about CCT.

**TABLE 2:** Outcomes of the studies that investigated the views of patients, significant others and mental health workers on the use of compulsory community treatment (CCT).

Stakeholder	Quantitative or qualitative	Country	Author	Year	Method	Participant	Experience with CCT	Summary of findings
**Patients**	Qualitative	USA	Scheid-Cook (3)	1993	Interviews	51 patients	Yes	Generally preferred CCT to admission
	Qualitative	England	Canvin et al. (16)	2002	Interviews	20 patients	Yes	Most believed CCT to be better than hospitalization
	Qualitative	Australia	Brophy and Ring (17)	2004	Focus groups	30 patients	Yes	Were generally dissatisfied with many aspects of CCT
	Qualitative	Canada	O’Reilly et al. (18)	2006	Interviews	14 patients	Yes	Preferred a CTO over returning to hospital
	Qualitative	England	Gault (19)	2009	Interviews	11 patients	No	All were opposed to CCT
	Qualitative	Canada	Schwartz et al. (20)	2010	Interviews	6 patients	Yes	Views on CCT were mixed
	Qualitative	Scotland	Ridley and Hunter (21)	2013	Interviews	49 patients	Partly (35%)	Welcomed CCT in the light of an alternative to involuntary admission
	Qualitative	England	Fahy et al. (22)	2013	Structured interviews	17 patients	Yes	Views on CCT were mixed
	Qualitative	Canada	Mfoafo-M’Carthy (23)	2014	Interviews	24 patients	Yes	Most participants expressed appreciation of CCT
	Qualitative	Norway	Riley et al. (24)	2014	Interviews	11 patients	Yes	Generally preferred CCT to admission
	Qualitative	Australia	Light et al. (25)	2014	Interviews	5 patients	Yes	Participants experienced ambivalence toward CCT
	Qualitative	England	Stroud et al. (26)	2015	Interviews	21 patients	Yes	Participants were often keen to stay on the CTO
	Qualitative	Norway	Stuen et al. (27)	2015	Interviews	15 patients	Yes	Participants had different views
	Qualitative	Norway	Stensrud et al. (28)	2015	Interviews	16 patients	Yes	Views on CCT were mixed
	Qualitative	England	Banks et al. (29)	2016	Interviews	21 patients	Yes	Most preferred CCT to admission
	Qualitative	Canada	O’Reilly et al. (30)	2016	Focus groups	20 patients	Yes	Were ambivalent about CCT
	Qualitative	Canada	Francombe et al. (31)	2018	Interviews	9 patients	Yes	Generally preferred CCT to admission
	Qualitative	Canada	Mfoafo-M’Carthy et al. (32)	2018	Interviews	11 patients	Yes	Most participants had negative feelings toward CCT
	Qualitative	England	Haynes and Stroud (33)	2019	Interviews	16 patients	Yes	Overall, patients saw CCT as more favorable than as adverse
	Qualitative	Australia	McMillan et al. (34)	2019	Interviews	8 patients	Yes	Participants had diverse experiences of CCT
	Qualitative	Australia	Brophy et al. (35)	2019	Interviews	8 patients	Yes	Most described CCT as wholly negative
	Qualitative	Australia	Dawson et al. (36)	2021	Interviews	8 patients	Yes	Some considered CCT to be benign; others felt it had been a negative experience
	Quantitative	USA	Swartz et al. (37)	2003	Interviews	123 patients	Yes	72% did not endorse the benefits of CCT
	Quantitative	USA	Swartz et al. (38)	2004	Interviews using vignettes	104 patients	Unclear	55% regarded CCT as fair, 62% as effective The majority preferred CCT to admission
	Quantitative	England	Crawford et al. (39)	2004	Structured interviews	103 patients	No	60% preferred CCT over admission
	Quantitative	New Zealand	Gibbs et al. (40)	2006	Semi structured interview	42 patients	Yes	65% was favorable toward CCT
	Quantitative	Ireland	O’Donoghue et al. (41)	2010	Interviews	67 patients	No	56% would prefer treatment in hospital to CCT
	Quantitative	New Zealand	Newton-Howes and Banks (42)	2014	Questionnaires	79 patients	Yes	53% thought they would have been better off treated informally
	Quantitative	Canada	Nakhost et al. (43)	2019	Interviews	69 patients	Yes	82% preferred CCT to admission
**Significant others**	Qualitative	USA	Swartz et al. (44)	2003	Interviews using vignettes	83 significant others	Unclear	Generally preferred CCT to admission
	Qualitative	Canada	O’Reilly et al. (45)	2006	Interviews focus groups	14 significant others	Yes	Were very positive about CCT
	Qualitative	New Zealand	Gibbs et al. (40)	2006	Semi-structured interviews	27 significant others	Yes	The great majority supported the use of CCT
	Qualitative	England	Gault (19)	2009	Interviews	8 significant others	No	All were opposed to CCT
	Qualitative	Australia	Light et al. (25)	2014	Interviews	6 significant others	Yes	Were ambivalent about CCT
	Qualitative	England	Stroud et al. (26)	2015	Interviews	7 significant others	Yes	Felt reassured and better consulted with CCT
	Qualitative	Norway	Stensrud et al. (46)	2015	Interviews	11 significant others	Yes	Generally supported the use of CCT
	Qualitative	Canada	O’Reilly et al. (30)	2016	Focus groups	18 significant others	Yes	Were positive about CCT
	Qualitative	England	Banks et al. (29)	2016	Interviews	7 significant others	Yes	Generally positive toward CCT
	Qualitative	England	Rugkasa and Canvin (47)	2017	Interviews	24 significant others	Yes	Generally supported the use of CCT
	Qualitative	Canada	Francombe et al. (31)	2018	Interviews	6 significant others	Yes	Generally preferred CCT to admission
	Qualitative	Australia	Brophy et al. (35)	2019	Interviews	30 significant others	Partly (33%)	Often identified the CTO as helping
	Quantitative	USA	McFarland et al. (48)	1990	Questionnaires	209 significant others	No	57% were in favor of outpatient commitment
	Quantitative	New Zealand	Vine and Komiti (49)	2015	Questionnaires	62 significant others	Partly (63%)	67% said that CTOs should be included in mental health legislation
**Mental health workers**	Qualitative	USA	Scheid-Cook (3)	1993	Interviews	73 mental health workers	Yes	Participants found CCT to be in general a good thing
	Qualitative	USA	Swartz et al. (44)	2003	Questionnaires with vignettes	85 mental health workers	Unclear	Generally preferred CCT to admission
	Qualitative	Canada	O’Reilly et al. (18)	2006	Focus groups	78 mental health workers	Yes	Most mental health workers felt that orders can be useful
	Qualitative	New Zealand	Gibbs et al. (40)	2006	Semi-structured interviews	90 mental health practitioners	Yes	Generally favored the use of CCT
	Qualitative	England	Taylor et al. (50)	2013	Questionnaires	9 mental health professionals	Yes	Participants were ambivalent about CCT
	Qualitative	USA	Sullivan et al. (51)	2014	Interviews	19 mental health workers	Yes	Participants were not unanimous in their comfort with CCT.
	Qualitative	England	Stroud et al. (26)	2015	Interviews	35 mental health workers	Yes	CTT was perceived helpful for certain patients
	Qualitative	Canada	O’Reilly et al. (30)	2016	Focus groups	27 mental health workers	Yes	Generally supported the use of CCT
	Qualitative	Norway	Stensrud et al. (52)	2016	Focus groups	22 mental health workers	Yes	Participants had a positive view of CCT
	Qualitative	Canada	Pridham et al. (53)	2018	Interviews	12 service providers	Yes	Saw CCT as a welcome alternative to admission
	Qualitative	Canada	Mfoafo-M’Carthy et al. (32)	2018	Focus group Interviews	6 mental health workers 1 psychiatrist, 1 programme coordinator	Yes	Believed that it was in the best interest of certain patients to use CCT
	Qualitative	Norway	Riley et al. (54)	2018	Interviews	9 mental health workers	Yes	Viewed CCT as a useful scheme
	Qualitative	Norway	Stuen et al. (55)	2018	Interviews Focus groups	8 clinicians 20 ACT-providers	Yes	Generally believed CTO’s were sometimes necessary
	Qualitative	England	Haynes and Stroud (33)	2019	Interviews	41 mental health professionals	Yes	Favored CCT over involuntary admission
	Qualitative	Australia	Brophy et al. (35)	2019	Interviews	30 mental health workers	Yes	Were ambivalent about CCT
	Quantitative	England	Burns (56)	1995	Questionnaires	59 psychiatrists 55 Community nurses 101 approved social workers	No	96% was willing to work with CCT 69% was willing to work with CCT 77% was willing to work with CCT
	Quantitative	Scotland	Atkinson et al. (57)	1997	Questionnaires	193 psychiatrists	No	86% were against CCT but did support the use of “leave of absence”
	Quantitative	England	Bhatti et al. (58)	1999	Structured interviews	83 mental health workers	No	68% supports the introduction of CCT
	Quantitative	England	Crawford et al. (59)	2000	Questionnaires	1171 psychiatrists	No	46% supported the use of CCT 35% were against 19% were unsure
	Quantitative	Scotland	Atkinson and Harper Gilmour (60)	2000	Questionnaires	230 psychiatrists 244 mental health officers	Partly (6%)	69% were against CCT 42% were against CCT
	Quantitative	Canada	O’Reilly et al. (61)	2000	Questionnaires	50 psychiatrists	Partly (48%)	62% is satisfied with the use of CCT
	Quantitative	United Kingdom	Pinfold et al. (62)	2002	Questionnaires	415 mental health workers	Might have	62% would not welcome powers of CCT
	Quantitative	Australia	Brophy and Ring (17)	2004	Interviews	18 mental health workers	Yes	72% was satisfied with the way the orders were used
	Quantitative	New Zealand	Romans et al. (63)	2004	Questionnaires	202 psychiatrists 82 mental health workers	Unclear	79% preferred to work in a system with CCT 85% preferred to work in a system with CCT
	Quantitative	USA	Christy et al. (64)	2009	Questionnaires	242 mental health workers	Partly (45%)	87% agreed with the use of CCT
	Quantitative	England and Wales	Manning et al. (65)	2011	Questionnaires	566 psychiatrists	Yes (most did)	60% preferred to work in a system with CCT
	Quantitative	England	Coyle et al. (66)	2013	Questionnaires	58 psychiatrists 212 other mental health workers	Unclear	83% supported the use of CCT 67% supported the use of CCT
	Quantitative	United Kingdom	Gupta et al. (67)	2015	Questionnaires	94 psychiatrists	Partly (78%)	55% stated that CCT helped to manage patients with complex needs
	Quantitative	Taiwan	Hsieh et al. (68)	2016	Questionnaires	176 mental health practitioners	Yes	75% preferred to work in a system with CCT
	Quantitative	Netherlands	De Waardt et al. (69)	2020	Interviews	40 mental health workers	Yes	73% supported the use of CCT
	Quantitative	Spain	Moleon Ruiz and Fuertes Rocanin (70)	2020	Interviews	32 psychiatrists, 10 residents [i.e., doctors] in psychiatry	No	92.8% supported the introduction of CCT
**Other**	Quantitative	USA	McFarland et al. (71)	1989	Questionnaires	92 commitment investigators 46 judges	No	72% supported the theory of outpatient commitment 74% supported the theory of outpatient commitment

Appendix 2 lists participants characteristics, the kind of service participants were recruited from and the available information about methods of recruitment.

### Data analysis and presentation

#### Patients

We found 29 manuscripts that reported on the views of patients, 22 of which were qualitative and seven of which were quantitative. Participants in 24 of the 29 studies had experience with CCT.

The studies were performed in eight different countries, being; Canada (*n* = 7), England (*n* = 7), Australia (*n* = 5), USA (*n* = 3), Norway (*n* = 3), New Zealand (*n* = 2), Scotland (*n* = 1), and Ireland (*n* = 1).

Of these 29 manuscripts, 14 found that the general opinion of patients was in favor of the use of CCT, eight found ambivalent views and seven found that the general opinion was against the use of CCT.

#### Significant others

In total, 14 manuscripts reported on the views of significant others (12 qualitative studies and 2 quantitative studies), 12 of them found that significant others supported the use of CCT, one found mixed feelings and one found that they were against the use of CCT.

In 11 of the 12 manuscripts in favor of CCT, the relatives had experience with CCT. So did the participants in the manuscript that reported mixed feelings. The participants in the manuscripts that found a negative attitude toward CCT did not have experience with CCT.

These manuscripts originated from six countries; England (*n* = 4), Canada (*n* = 3), New Zealand (*n* = 2), USA (*n* = 2), Australia (*n* = 2), and Norway (*n* = 1).

#### Mental health workers

Of the 31 manuscripts that reported the views of mental health workers (15 qualitative and 16 quantitative studies), 24 found that the majority of mental health workers supported the use of CCT, 4 found their participants to have mixed feelings and 3 found that their participants were mainly against the use of CCT. Two out of three studies in this last group were carried out in Scotland around the time CCT was implemented; the participants in these studies did not have experience with CCT.

These studies were performed in 13 different regions/countries: England (*n* = 7), Canada (*n* = 5), USA (*n* = 4), Norway (*n* = 3), New Zealand (*n* = 2), Australia (*n* = 2), Scotland (*n* = 2), United Kingdom (*n* = 2), Taiwan (*n* = 1), the Netherlands (*n* = 1), England and Wales (*n* = 1), and Spain (*n* = 1).

There was a wide range of different mental health workers who participated in the studies, amongst them were psychiatrists, psychologists, nurses, social workers, and occupational therapist. Appendix 2 lists the specific occupations for each study, as far as they were reported.

We found no manuscripts that reported the views of policy makers; we did find one study on the views of judges and commitment investigators, the majority of whom supported the use of CCT.

Overall, there are more studies that reported that patients were against the use of CCT (7 out of 29 studies), compared to relatives (1 out of 14 studies) and mental health workers (3 out of 31 studies).

But all stakeholder groups report ambivalence toward CCT.

Since most studies concerned stakeholders with experience, no conclusion can be drawn for all stakeholder groups regarding the influence of experience with CCT on the opinion on CCT.

The majority of these studies (67%) obtained qualitative data and only 18 (33%) studies obtained quantitative data. The 18 quantitative studies used different outcome measures, such as preferring to work in a system using CCT, or stating that CCT helps patients with complex needs.

Table 3 lists the advantages and disadvantages of CCT mentioned by stakeholders in the various studies. These are ranked from mentioned in most manuscripts to mentioned in least.

**TABLE 3:** The five advantages/disadvantages reported most often in studies of experience and views of compulsory community treatment (CCT).

Advantages
Patients
- CCT facilitated access to care - Patients experienced increased support - CCT could improve mental health - CCT provided more freedom than involuntary admission - CCT provided a safety net and a sense of security
Significant others
- CCT facilitated access to care - CCT facilitated earlier admission - CCT could provide more safety for the patient - CCT could take some of the burden away from family members - CCT could lead to greater carer involvement
Mental health workers
- CCT provided an opportunity to stay in touch and to monitor the patient’s mental health - CCT could enhance compliance to treatment - CCT could provide a safety net - Provided more freedom than involuntary admission - CCT could improve mental health and avoid involuntary admission

**Disadvantages**

Patients
- CCT constrained autonomy and was coercive - CCT was stigmatizing - CCT interfered with daily life - The focus of CCT lay too much on medication - Patients had to deal with the side-effects of forced medication
Significant others
- CCT constrained autonomy and was coercive - CCT focused too much on medication - The process of applying for CCT was too cumbersome - CCT could be stigmatizing - CCT also put a strain on carers, involving them in treatment
Mental health workers
- CCT constrained autonomy and is coercive - CCT could interfere with the therapeutic relationship - CCT imposed an extra administrative burden - CCT could be stigmatizing - CCT focused too much on medication

The advantage mentioned most often for all stakeholder groups was that CCT facilitated access to care. Furthermore, patients mentioned that they experienced increased support in case of CCT versus not having CCT. Significant others expressed that CCT facilitated earlier admission as an important advantage. And for mental health workers a great advantage was also that it could enhance compliance with treatment.

The most mentioned disadvantage by all stakeholder groups was that CCT restricted autonomy and was coercive. Patients mentioned as second most often that it was stigmatizing. For significant others the second most often mentioned disadvantage was that it focused too much on medication and for mental health workers the second most often mentioned disadvantage was that CCT sometimes interfered with the therapeutic relationship.

## Discussion

Despite the lack of scientific evidence for the effects of CCT, this integrative review showed that in half of the studies patients, and in the majority of the studies significant others and mental health workers favored a mental health system that included CCT. Nonetheless, nearly all studies indicated that stakeholders expressed ambivalences about CCT. Patients were more critical regarding the use of CCT than the other stakeholders. The question remains why, despite the ambivalence it raised and in the absence of empirical evidence of its effectiveness, CCT is implemented in so many countries.

It can be helpful to look at the advantages as well as the disadvantages of CCT mentioned by stakeholders more in detail.

The advantage of CCT mostly indicated by patients and significant others was that it facilitates access to care. The rationale for this may be that, if a patient’s situation deteriorated (when being on CCT) he or she would always have someone to contact who could provide the necessary (inpatient) care. The most valued advantage of CCT for mental health workers was that it provided a way to monitor a patient’s health and stay in touch with the patient. This improved access to care is supported in some uncontrolled studies that found that CCT increased the number of outpatient contacts (6).

Another advantage frequently mentioned, was that it provides a safety net and a sense of security.

Research findings also suggest that CCT could provide more safety, since there are studies that find that people on a CTO have a lower mortality rate (10), have lower suicide numbers (11) and were more likely to receive acute medical care for a physical illness (72).

The fact that these advantages seem to be so important for the stakeholders, is an interesting finding, as these advantages also could be achieved without CCT, as long as there is adequate access to care and continuity of care. – as in Italy, where outpatient care is easily accessible (73).

However, it has been argued that just the availability and accessibility of mental health care services alone is not enough to engage all groups of patients into mental health care (30).

The disadvantages mentioned mostly by all stakeholders were that CCT is a coercive measure that it constrains autonomy, and also that it is stigmatizing. Some authors argue on the other hand that CCT can help patients regain their autonomy - and reduces stigma when their stability improves (2). Another disadvantage all stakeholder groups mentioned, is the excessive focus on taking medication. Studies into the main reasons for deciding on using a CTO for mental health workers show that adherence to treatment is the most important reason for deciding to use a CTO (63, 65). Maybe this is because medication is something that mental health workers can easily provide (in contrast to proper housing or daytime activities) and it has proven to be effective in improving certain symptoms of mental health disorders. However, in a study on the opinions of mental health workers, mental health workers stressed that treatment not only involves medication, but other factors were also essential, such as a good therapeutic relationship, proper housing and access to jobs or daytime activities (69).

Overall we find that the majority of the stakeholders prefer a system with CCT and apparently puts the emphasis on the advantages, accepting the disadvantages. Corring et al. (12) come to a similar conclusion in their comparative analysis.

When interpreting studies about the opinions of stakeholders on CCT, it should be kept in mind that there is a difference between comparing CCT with involuntary admission and comparing it with voluntary care in the community. A patient could prefer CCT to hospitalization, but if there was the choice between voluntary care in the community or CCT, this person might choose voluntary care. They thus seem to support CCT, but only if the alternative were hospitalization. In many of the studies in which patients reported that they supported CCT, they meant that they preferred it to admission to hospital.

We think patients’ preference should be taken into account when deciding on compulsory care. This practice is already in place in the Netherlands in the new Dutch mental health legislation in which patients make a care plan which entails that patients have the opportunity to state their preferences regarding compulsory care.

O’Reilly et al. describe a general consensus that “the use of CTO”s is justifiable for certain individuals, but only if it can be shown that CTOs confer significant benefits on those individuals’ (74) which leaves room for patients and their mental health care workers to decide to use CCT if they think it helps the patient.

### Strengths

The main strength of this integrative review is that it included quantitative as well as qualitative studies. Another strength is that in the literature search we did not focus on specific stakeholder groups but were open for views of all relevant groups.

### Limitations

The review protocol was not prospectively registered, however, no protocol changes have been made during the process, also no separate study quality appraisal has been performed for all the studies included.

Many of the studies included in this review were qualitative studies that were not designed to report representative views, but rather to provide the breadth and nuance of experiences in this field. Views on CCT are all very complex and almost always ambivalent, this makes it difficult to state whether participants are “pro or con” CCT. For that reason we also explicitly investigated the advantages and disadvantages reported in these studies.

Also there might be a form of selection bias, since most of the patient participants were recruited through their mental health workers or they signed up for the study themselves. This could mean that the patients who were doing well or were more satisfied with their treatment, were more likely to participate in the studies.

## Implications for future research

First, it remains important to investigate further why stakeholders would support CCT. If accessibility and continuity of care is one of the main reasons, countries should invest in accessible voluntary care and further studies should be done to see how we can engage patients more easily in voluntary care rather than relying on coercive legal structures. Second, it would be good to include policymakers and other stakeholders, like judges or general practitioners in this research, in order to investigate the grounds on which mental health laws on CCT are developed and implemented.

## Conclusion

While the majority of all stakeholders appears to support the use of CCT, many have reservations. Stakeholders considered the most important advantages of CCT to be access to care and a way to remain in contact with patients and monitor their health, especially during times of crisis or deterioration. Stakeholders mention as the most serious disadvantage the restrictions CCT imposes on patients’ freedom and autonomy, stigmatization, and the focus on the use of medication.

## Author contributions

DW wrote the research plan, performed the literature analysis, and wrote the first version and later versions of the manuscript. AM performed the literature analysis and contributed to the manuscript. WB developed the literature search, wrote part of the methodology section, and contributed to the manuscript. FH worked on the initial research plan and contributed to the manuscript. JR worked on the analysis of the data and contributed to the manuscript. GW and CM worked on the research plan, the analysis of the data, and took part in writing the manuscript. All authors contributed to the article and approved the submitted version.
