# Supplementary material for: Use of compulsory community treatment in mental healthcare: An integrative review of stakeholders’ opinions
Source: Front Psychiatry. 2022 Nov 3;13:1011961. doi: 10.3389/fpsyt.2022.1011961 (PMC9669570; doi:10.3389/fpsyt.2022.1011961)
Supplement: Supplementary file 1 [file Table_1.DOCX]

Appendix 1

Databases and search terms used in the literature search

| Database | Search terms |
| --- | --- |
| embase.com | ('compulsory treatment'/de OR 'involuntary commitment'/de OR 'persuasive communication'/de OR 'community treatment order'/de OR (compulsor* OR involuntar* OR coerci* OR commitment* OR forcib* OR community-treatment-order*):ab,ti) AND (outpatient/de OR 'outpatient care'/de OR 'community care'/de OR 'community'/de OR 'community mental health'/de OR 'ambulatory care'/exp OR 'community treatment order'/de OR (outpatient* OR communit* OR ambulator*):ab,ti) AND ('mental health care'/exp OR 'mental health'/exp OR 'psychiatric treatment'/exp OR 'psychiatry'/exp OR 'mental patient'/exp OR 'mental disease'/exp OR 'community treatment order'/de OR 'community mental health'/de OR 'mental health care personnel'/exp OR (mental* OR psychiatr* OR community-treatment-order*):ab,ti) AND ('experience'/exp OR 'personal experience'/exp OR perception/de OR 'health personnel attitude'/exp OR (experienc* OR view OR views OR preference* OR opinion* OR perception* OR attitude*):ab,ti) NOT ([Conference Abstract]/lim) AND [English]/lim |
| Medline ovid | (Involuntary Treatment/ OR Commitment of Mentally Ill/ OR Persuasive Communication/ OR (compulsor* OR involuntar* OR coerci* OR commitment* OR forcib* OR community-treatment-order*).ab,ti.) AND (Ambulatory Care Facilities/ OR Outpatients/ OR Community Health Services/ OR Community Psychiatry/ OR Ambulatory Care/ OR Community Mental Health Services/ OR (outpatient* OR communit* OR ambulator*).ab,ti.) AND (Mental Health Services/ OR exp Mental Health/ OR exp Psychiatry/ OR Mentally Ill Persons/ OR exp Mental Disorders/ OR Community Psychiatry/ OR Community Mental Health Services/ OR (mental* OR psychiatr* OR community-treatment-order*).ab,ti.) AND (Perception/ OR exp Attitude of Health Personnel/ OR (experienc* OR view OR views OR preference* OR opinion* OR perception* OR attitude*).ab,ti.) NOT (news OR congres* OR abstract* OR book* OR chapter* OR dissertation abstract*).pt. AND english.la. |
| PsycINFO ovid | (Involuntary Treatment/ OR "Commitment (Psychiatric)" OR coercion/ OR (compulsor* OR involuntar* OR coerci* OR commitment* OR forcib* OR community-treatment-order*).ab,ti.) AND (Outpatient Treatment/ OR Outpatients/ OR Community Services/ OR Community Psychiatry/ OR Community Mental Health Services/ OR (outpatient* OR communit* OR ambulator*).ab,ti.) AND (Mental Health Services/ OR exp Mental Health/ OR exp Psychiatry/ OR Psychiatric Patients/ OR exp Mental Disorders/ OR Community Psychiatry/ OR Community Mental Health Services/ OR (mental* OR psychiatr* OR community-treatment-order*).ab,ti.) AND (Perception/ OR exp Health Personnel Attitudes/ OR (experienc* OR view OR views OR preference* OR opinion* OR perception* OR attitude*).ab,ti.) NOT (news OR congres* OR abstract* OR book* OR chapter* OR dissertation abstract*).pt. AND english.la. |
| CINAHL EBSCOhost | (MH Involuntary Treatment OR MH Involuntary Commitment OR MH Persuasive Communication OR TI (compulsor* OR involuntar* OR coerci* OR commitment* OR forcib* OR community-treatment-order*) OR AB (compulsor* OR involuntar* OR coerci* OR commitment* OR forcib* OR community-treatment-order*)) AND (MH Ambulatory Care Facilities OR MH Outpatients OR MH Community Health Services OR MH Ambulatory Care OR MH Community Mental Health Services OR TI (outpatient* OR communit* OR ambulator*) OR AB (outpatient* OR communit* OR ambulator*)) AND (MH Mental Health Services+ OR MH Mental Health+ OR MH Psychiatry OR MH Mental Disorders+ OR MH Community Mental Health Services OR TI (mental* OR psychiatr* OR community-treatment-order*) OR AB (mental* OR psychiatr* OR community-treatment-order*)) AND (MH Perception OR MH Attitude of Health Personnel OR TI (experienc* OR view OR views OR preference* OR opinion* OR perception* OR attitude*) OR AB (experienc* OR view OR views OR preference* OR opinion* OR perception* OR attitude*)) NOT PT (news OR congres* OR abstract* OR book* OR chapter* OR dissertation abstract*) AND LA(English) |
| Web of science Core Collection | TS=(((compulsor* OR involuntar* OR coerci* OR commitment* OR forcib* OR community-treatment-order*)) AND ((outpatient* OR communit* OR ambulator*)) AND ((mental* OR psychiatr* OR community-treatment-order*)) AND ((experienc* OR view OR views OR preference* OR opinion* OR perception* OR attitude*))) AND DT=(article) AND LA=(english) |
| Cochrane CENTRAL register of trials | ((compulsor* OR involuntar* OR coerci* OR commitment* OR forcib* OR community-treatment-order*):ab,ti) AND ((outpatient* OR communit* OR ambulator*):ab,ti) AND ((mental* OR psychiatr* OR community-treatment-order*):ab,ti) AND ((experienc* OR view OR views OR preference* OR opinion* OR perception* OR attitude*):ab,ti) |
| Google scholar | compulsory\|involuntary\|coercive\|coercion\|commitment\|forcible outpatient\|community\|ambulatory mental\|psychiatry\|psychiatric experiences\|views\|preferences\|opinions\|perceptions\|attitudes |
