# Supplementary material for: Use of compulsory community treatment in mental healthcare: An integrative review of stakeholders’ opinions
Source: Front Psychiatry. 2022 Nov 3;13:1011961. doi: 10.3389/fpsyt.2022.1011961 (PMC9669570; doi:10.3389/fpsyt.2022.1011961)
Supplement: Supplementary file 2 [file Table_2.DOCX]

| **Author** | **Year** | **Characteristics of participants** | **Service setting** | **Recruitment method** |
| --- | --- | --- | --- | --- |
| Atkinson (57) | 1997 | 193 consultant psychiatrists | Adult general psychiatry in Scotland | Questionnaires were sent out |
| Atkinson  (60) | 2000 | 474 mental health workers  Occupation: 230 consultant psychiatrists,  244 social workers with mental health officer status | Adult general psychiatry in Scotland | Questionnaires were sent out |
| Banks  (29) | 2016 | 21 service users  Gender: 67% male, 33% female  Mean age: 39 years  Diagnosis including schizophrenia, schizoaffective disorder and bipolair disorder | A particular NHS trust (not further specified) | Purposive sampling |
|  |  | 7 relatives  Gender: 2 male, 5 female |  |  |
| Bhatti  (58) | 1999 | 83 mental health workers  Occupation: 35 consultant psychiatrists, 14 senior or specialist registrars, 10 senior clinical medical officers, 9 clinical assistants, 5 associate specialists, 10 general practitioners  Mean period they had been approved: 9 years | Section 12(2)-approved practioners in the West Midlands, England | A random sample was approached of which 83 practitioners agreed to participate |
| Brophy  (35) | 2019 | 8 service users with experience of being on a CTO  30 relatives | People living in Victoria with experience of a mental health illness | Through posters, staff contact and email networks |
|  |  | 30 mental health workers  Occupation: 10 psychiatrists, 20 mental health practitioners | Clinical and non-clinical services in Victoria | Information distributed at sector events and through professional associates |
| Brophy  (17) | 2004 | 30 service users  Gender: most likely to be male  Age: most likely in their early 40s  Diagnosis: most likely schizophrenia | People living in Victoria with experience of a mental health illness and outpatient services | Unclear |
|  |  | 18 mental health workers  Gender: mostly women  Occupation: 6 social workers, 2 psychiatrists/medical officers, 2 occupational therapists, 2 lawyers, 2 psychiatric nurses, 1 medical records officer, 1 advocate, 1 solicitor, 1 welfare worker | A range of psychiatric services in Victoria | Randomly selected via telephone request |
| Burns  (56) | 1995 | 215 mental health workers  Occupation: 59 psychiatrists, 55 Community nurses, 101 approved social workers | All general, forensic and old-age consultant psychiatrists employed in South West Thames | Questionnaires were mailed |
| Canvin  (16) | 2002 | 20 service users;  Gender: 14 male, 6 female  Age range: 20-74 years | Outpatient services in the south-east of England | Participants were contacted through their key worker |
| Christy  (64) | 2009 | 242 mental health workers | Different mental health services both in- and outpatient | Online survey, link was sent to people who had attended a specific training as well as to contacts at a variety of providers |
| Coyle  (66) | 2013 | 286 mental health workers  Gender: 38% male, 62% female  Age: 63% was aged between 31 and 50 years  Occupation: 59 doctors, 20 team managers, 95 nurses, 52 social workers and 60 ‘other occupations’ | Adult community mental health teams in 2Gether and Oxford Health NHS Foundation Trusts | All medical and non-medical staff were invited to complete the survey |
| Crawford  (59) | 2000 | 1171 consultant psychiatrists working in England or Wales | Could be any setting | Postal Survey |
| Crawford  (39) | 2004 | 109 patients  Gender: 64 male, 45 female  Diagnosis: psychosis (n=109), neurosis (n=15), alcohol/drugmisuse (n=9), personality disorder (n=12) | Patients discharged from a mental health unit in West London | All patients who had been admitted were approached |
| Dawson  (36) | 2021 | 8 service users,  Gender: 7 male, 1 female  Age range: 19-49 years (median = 40).  Contact with mental health services ranging from 5-20 years. | Two community mental health teams in Adelaide, South Australia | Clinicians approached service users |
| De Waardt  (69) | 2020 | 40 mental health workers  Gender: 23 male, 17 female  Age range: 30-64 years  Occupation: 30 psychiatrists, 10 community nurses  Mean number of years of experience on the job: 9 years | Community mental health teams and inpatient facilities | Different mental health institutions were approached, inviting mental health workers to participate |
| Fahy  (22) | 2013 | 17 patients  Gender: 14 male, 3 female.  Age range: early 20s to mid 60s.  Diagnose: primary diagnosis of schizophrenia (n=12), 3 schizo-affective disorder (n=3), delusional disorder (n=1), mental and behavioural disorder secondary to alcohol (n=1) | One Early Intervention Team and one Assertive Outreach Team in Merseyside England | Patients were recruited through their care co-ordinators |
| Francombe  (31) | 2018 | 9 patients,  Age range: 20-60 years  Time they had been in contact with a mental health service: 2-39 years | Three community mental health teams in Toronto, Canada | Patients and their relatives were approached by their mental health worker.  Mental health workers were directly approached by the researcher |
|  |  | 6 relatives  Relationship: 5 were parents of a patient, one was a spouse |  |  |
|  |  | 12 mental health workers  Age: 26-66.  Occupation: 5 nurses, 4 occupational therapists, 2 psychiatrists, 1 social worker  Time working in their current team: from 1 to 15 years. |  |  |
| Gault  (19) | 2009 | 11 patients | Participants were accessed through local support groups, NHS, England. | Patients volunteered for the study and could involve their carer. |
|  |  | 8 carers |  |  |
| Gibbs  (40) | 2006 | 42 patients  Gender: 32 male, 10 female  Mean age: 38.  Diagnosis: Schizophrenia (n = 23), Affective psychosis (n = 10), Schizoaffective (n = 7), Personality disorder (n = 1) and other (n = 1). | Outpatient service, Otago, New Zealand | Patients were approached when their key worker had assessed their capacity to participate. Relatives were nominated by the patients. And the involved key worker and psychiatrist were included. |
|  |  | 27 relatives |  |  |
|  |  | 90 mental health workers |  |  |
| Gupta  (67) | 2015 | 94 psychiatrists specialized in ID  78 % had used CTO’s before | Community Intellectual Disability teams, UK | A survey was sent out via e-mail to all registered ID-psychiatrists. |
| Haynes  (33) | 2019 | 16 service users | Unclear | Unclear |
|  |  | 41 mental health workers;  Occupation: 17 responsible clinicians (usually psychiatrists), 24 care co-ordinators (16 community nurses, 7 social workers and one occupational therapist). |  |  |
| Hsieh  (68) | 2017 | 176 mental health workers  Gender: 64 male, 112 female, 4 gender unknown.  Age range: 38 – 69 years  Mean clinical experience: 18 years | Unclear | Surveys were sent out |
| Light  (25) | 2014 | 5 patients | Unclear | Purposive sampling, participants volunteered for the study. |
|  |  | 6 carers |  |  |
| Manning  (65) | 2011 | 566 psychiatrists  Gender: 66 % male, 34% female  Clinical experience: 73 % had worked as a psychiatrist for 10 – 30 years. | Could be any service in England or Wales | Postal survey |
| McFarland  (71) | 1989 | 92 investigators  Gender: 51 male, 41 female  Mean age: 40 years  Average years on the job: 7 years  46 judges  Gender: 46 male  Mean age: 52 years  Average years on the job: 11 years | Oregon Circuit court and members of the Commitment Investigators Association of Oregon | Postal survey |
| McFarland  (48) | 1990 | 260 relatives | Probably outpatient services in Oregon, USA. | A questionnaire was distributed through mental health workers and through the Alliance of Advocates for mentally ill |
| McMillan  (34) | 2019 | 8 patients, currently on CTO | Clinical mental health services, Adelaide, Australia | Patients were recruited via their mental health care workers |
| Mfoafo-M’Carthy  (23) | 2014 | 24 patients,  Gender: 9 male, 15 female  Age range: 18-59 years  Had been diagnosed with mental illness between 1 to 17 years previously.  Diagnosis: schizophrenia (n = 16), schizo-affective (n = 1), depression (n = 2) and bipolair disorder (n = 5) | Either community teams or hospitals in Toronto, Canada | Recruited through information flyers at several places in the community and from hospitals |
| Mfoafo-  M’Carthy  (32) | 2018 | 11 patients  Gender: 6 male, 5 female  Age range 27-66 years (mean 40.5)  Involved with the mental health system between 4 and 51 years, (mean 16.3)  Diagnosis: bipolar disorder (n=7), schizophrenia (n=3), drug induced psychosis (n=1) | ACT team in Southwestern Ontario Canada | Patients were approached by their mental health worker |
|  |  | 8 mental health workers  Occupation: 6 mental health workers,  1 psychiatrist, 1 programme coordinator |  | Clinicians were recruited through information letters |
| Moleón Ruiz  (70) | 2020 | 42 mental health workers  Occupation: 32 psychiatrists, 10 resident medical interns in psychiatry. | Psychiatric Service of the University Hospital Complex of Huelva, Spain | Participants were personally invited to participate |
| Nakhorst  (43) | 2019 | 69 patients  Gender: 43 male, 26 female  Mean age: 38.4 years  Most had a diagnosis of psychotic disorder (n = 58) | Outpatient services from 3 mental health centres in the city of Toronto, Canada | Patients were informed about the study by their community mental health teams |
| Newton-Howes  (42) | 2014 | 79 patients  Gender: 58% male, 42% female  Mean age: 42 years  Diagnosis: schizophrenia (58%), bipolar disorder (17%), other psychotic disorders (11%). | Secondary care services in New Zealand | All potential participants were approached and asked to participate. |
| O’Donoghue  (41) | 2010 | 67 patients  Gender: 33 male, 34 female  Age range males: 18-67 years (mean 37)  Age range females: 22-77 years (mean 45)  Diagnosis: schizophrenia (n = 38), affective disorders (n = 24), other (n = 5) | Clinical ward of St John of God Hospital, Dublin, Ireland | Patients were approached by the research team and invited to participate |
| O’Reilly  (61) | 2000 | 50 psychiatrists  Gender: 73 % male, 27% female  Clinical experience: range: 1-49 years, mean 17 years  Specialty: 68% practiced general adult psychiatry, 10 % child psychiatry and 10 % forensic psychiatry | Psychiatrists registered to practice in Saskatchewan, Canada | Postal survey |
| O’Reilly  (18) | 2006 | 14 patients  Age range 20-70 years (mean 44)  Diagnosis: schizophrenia (n = 9), schizoaffective disorder (n = 5) | Two out patient services in Saskatchewan, Canada | Patients were identified and approached by their mental health workers |
|  |  | 14 family members  78 mental health workers from a variety of backgrounds, majority were nurses |  | Participants were invited to attend focusgroups and a snowball sample of convenience was used |
| O’Reilly  (30) | 2016 | 20 patients:  Gender: 55% male, 45% female  Mean age: 43 years  Diagnosis: Schizophrenia (n = 10), Schizoaffective disorder (n = 6), Bipolar disorder (n = 3), other psychotic disorder ( n = 1) | 7 ACT teams in London, Canada | Patients who were deemed able to consent to participate by their psychiatrist were selected randomly by the research team and were invited to participate |
|  |  | 18 relatives  Gender: 4 male, 14 female |  | The participating relatives were relatives of the participating patients |
|  |  | 27 ACT clinicians  Gender: 33% male, 67% female  Occupation: Nurses (63%), Social workers (19%), Occupational Therapists (15%), Vocational counselers (4%)  Mean time on a ACT team: 6.9 years |  | Unclear how ACT clinicians were recruited for the focusgroups. |
| Pinfold  (62) | 2002 | 415 mental health workers  Gender: 191 male, 224 female  Aged under 50: 80%  Occupation: Consultant psychiatrist (n=77), Community mental health team leaders (n=57), community psychiatric nurse (n=147), mental health social worker (n=83) | 12 mental health provider Trusts and associated Local Authorities (LAs) in England | Questionnaires were sent out |
| Ridley  (21) | 2013 | 49 patients  Gender: 67% male, 33% female  Age range: 21 – 63 years (mean 40.5) | Both out patient NHS services and two hospitals | Purposive sampling, patients volunteered to participate |
| Riley  (24) | 2014 | 11 patients  Gender: 7 male, 4 female  Age range: 23 – 60 years  Diagnosis: schizophrenia, schizotypal and delusional disorders | Outpatients treated at University Hospital of North Norway | Purposive sampling, patients were approached through their mental health worker |
| Riley  (54) | 2018 | 9 mental health workers  Occupation: 2 specialist clinical psychologists, 7 psychiatrists  Each had several years of experience as CTO decision makers | University Hospital of North Norway | Participants were approached by the researcher when their patients were participating in another study |
| Romans  (63) | 2004 | 202 respondents in the national psychiatrist survey  Gender: 61% male, 39% female, one with gender missing  82 respondents in the regional survey of other MHPs.  Gender: 40% male, 52% female, six with gender missing  Occupation: nurses were the largest occupational category in the MHP group (n = 35). | Physicians registered with the NZ Medical Council as specialists in psychiatry in 2000 and community-based, publicly employed, mental health professionals (MPHs) in the province of Otago | A postal survey was sent out |
| Rugkasa  (47) | 2017 | 24 carers  Relationship: mostly parents of patients on CTO’s, one was a sister and one was a husband. | Outpatient services all over England | Participants signed up for the study |
|  |  | 73 clinicians  Gender: majority were women  Occupation: degrees in social work, psychology or nursing |  |  |
| Taylor  (50) | 2013 | 9 approved mental health professionals | Local social services authority in the north west of England | A questionnaire was sent out |
| Scheid-Cook  (3) | 1993 | 51 clients | Outpatient services in North-Carolina, U.S.A. | Unclear |
|  |  | 73 clinicians  Gender: majority were women  Occupation: social workers, psychologists and nurses |  |  |
| Schwartz  (20) | 2010 | 6 patients  Gender: 5 males, 1 female  Ages range: early 20s to mid 50s  Length on the community treatment order ranged from four months to seven years | Mental health centers in Ottawa, Canada | Participants were approached by their CTO coordinator and asked to participate |
| Stensrud  (28) | 2015 | 16 patients  Gender: 8 male, 8 female  Age range: 26-66 years (median 43)  Diagnosis: schizophrenia (n=12), affective disorder (n=3), another psychiatric disorder (n=1)  Duration of contact with mental health services: 3 to 26 years, median 11.5 years | Outpatient services in two counties in Eastern Norway | Patients were approached and invited through their psychiatrist |
| Stensrud  (46) | 2015 | 11 relatives  Gender: 3 male, 8 female  Age range: 20 - 83 years  Relationship: four parents, four siblings, one wife, one daughter and one brother’s wife | Outpatient services in two counties in Eastern Norway | Participants were invited to participate when their relative consented to them being approached |
| Stensrud  (52) | 2016 | 22 mental health workers  Gender: 9 men, 13 women | One hospital, four districts psychiatric centers and three local authorities in Eastern Norway | Participants were invited through an invitation sent to their head of department |
| Stroud  (26) | 2015 | 21 service users  Gender: 67% male, 33% female  Mean age: 36  Diagnosis: schizophrenia, schizoaffective disorder, bipolar affective disorder | Mental health NHS trust in Southern England | Patients were invited to participate through their care coordinators. |
|  |  | 7 relatives  Gender: 2 male, 5 female |  | Relatives were contacted by Trust administrators |
|  |  | 35 mental health professionals  Occupation: 16 care coordinators, 10 responsible clinicians, 9 AMHPS |  | Practitioners were contacted directly, purposive sampling was used |
| Stuen  (27) | 2015 | 15 patients  Gender: 9 male, 6 female  Age: 5 patients younger than 37, 6 patients aged between 37 and 47, 4 patients older than 47  Diagnosis: psychotic disorders (n=10), schizoaffective disorder (n=5) | 4 ACT teams in Norway | Patients were approached to participate through their mental health workers |
| Stuen  (55) | 2018 | 28 mental health workers  Occupation: 6 psychiatrists, 2 clinical psychologists, 3 psychologists, 8 psychiatric nurses, 2 social educators, 4 social workers, 1 nursing assistant, 2 peer specialists | 4 ACT teams in Norway | Participants were invited to participate |
| Sullivan  (51) | 2014 | 19 mental health workers  Occupation: most were social workers or psychologists  Average time on the job: 8 years | Two community mental health centers in a metropolitan area in the Midwest, USA. | Not entirely clear. It seems participants signed up to participate. |
| Swartz  (37) | 2003 | 123 patients  Diagnosis: schizophrenia, schizoaffective disorder, other psychotic disorder or major affective disorder | Outpatient services in North Carolina, USA | Patients were approached during their admission in hospital |
| Swartz  (44) | 2003 | 83 family members  Gender: 21 male, 62 female  Mean age: 54 years | Outpatient services in North Carolina, USA | Patients who were approached for this study were asked if their relatives could be approached too. (Patient outcomes art described in Swartz 2004) |
|  |  | 85 clinicians:  Gender: 34 male, 51 female  Mean age: 44 years  Mean number of years of experience on the job: 13 years |  | Clinicians were selected through their employers and received a survey via mail |
| Swartz  (38) | 2004 | 104 patients,  Gender: 57 male, 47 female  Mean age: 44 years  Diagnosis: schizophrenia, schizoaffective disorder, schizophreniform disorder | Outpatient services in North Carolina, USA | Patients were approached after participating in another study |
| Vine  (49) | 2015 | 62 relatives  Gender: 10 %, 90% female  Mean age: 63 years  Relation: 82% were the parent of a person with mental illness | Two carer support organisations in Australia | Carers were approached through two carer support organisations and received a postal survey |
